# Supplementary figures and images for: Disruption of the nascent polypeptide-associated complex leads to reduced polyglutamine aggregation and toxicity
Source: PLoS One. 2024 Aug 15;19(8):e0303008. doi: 10.1371/journal.pone.0303008 (PMC11326622; doi:10.1371/journal.pone.0303008)

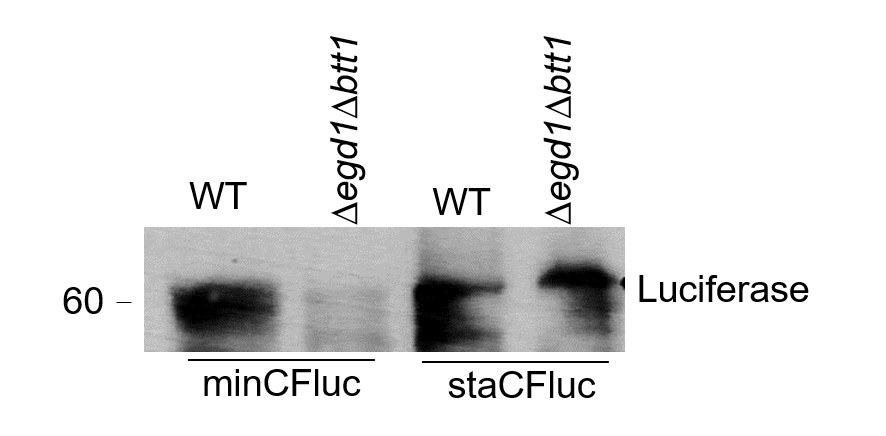

Supplement: S1 Fig — Yeast 74-D694 WT and NAC deletion strains expressing minCFLuc, and RLuc codon variants from centromeric (single copy) plasmids using identical transcriptional and translational control sequences, consisting of the transcriptional promoter and 5′-UTR of the yeast TDH3 (glyceraldehyde-3-phosphate dehydrogenase, GPD) gene, and of the 3′-UTR and transcriptional terminator sequences of the yeast ADH1 (alcohol dehydrogenase) gene. Both TDH3 and ADH1 are highly expressed endogenous yeast genes. These are grown in SD-Ura broth and lysed using standard yeast lysis protocol for western blot analysis using Luciferase antibody. (TIF) [file pone.0303008.s001.tif]

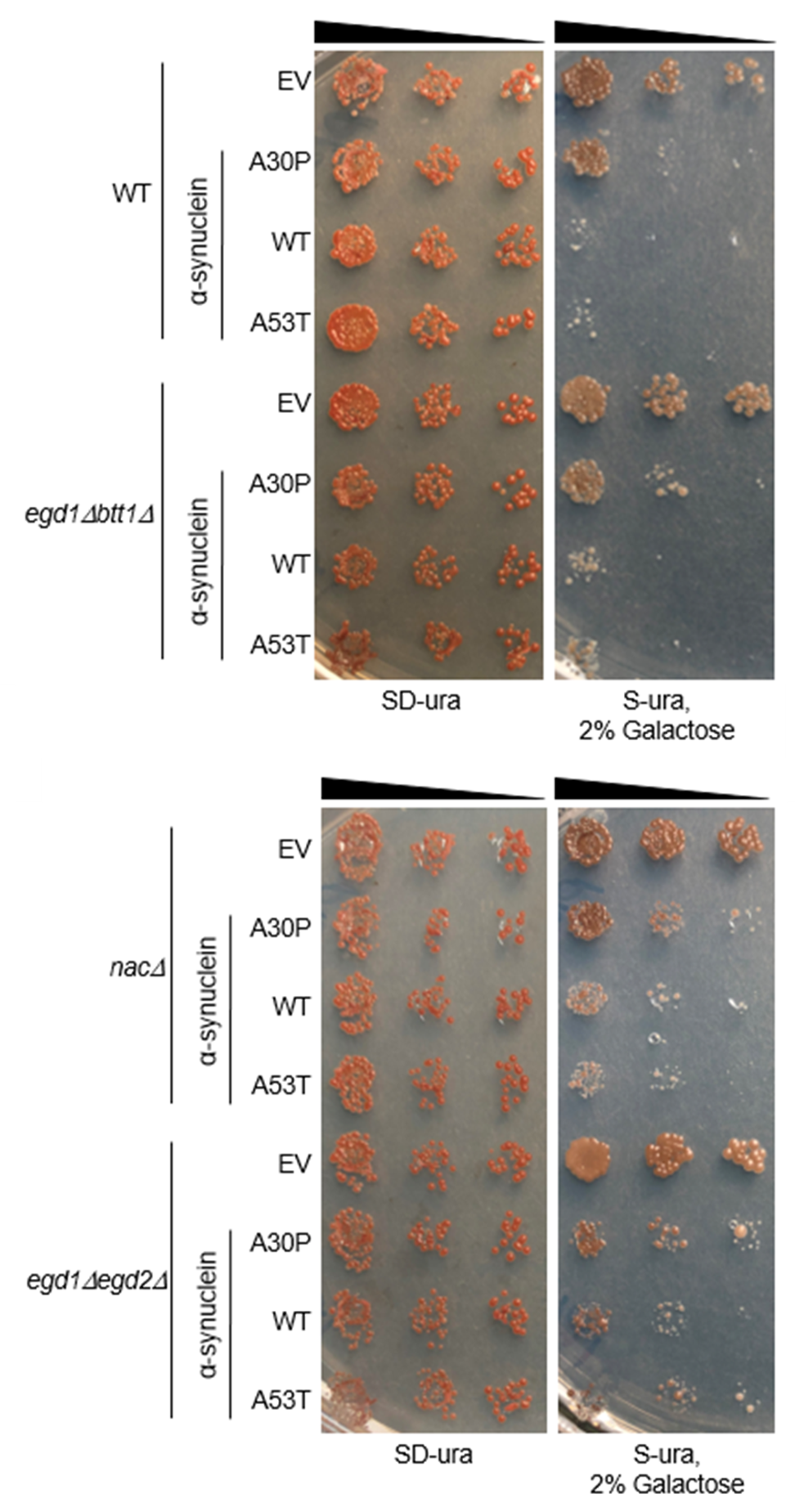

Supplement: S2 Fig — Yeast 74-D694 WT and nac deletion strains expressing Gal1-inducible EV or WT, A30P, or A53T α-synuclein constructs were serially diluted 5-fold and spotted onto ¼ YPD (not shown), SD-ura, and S-ura, 2% galactose (first 3 spots shown) to monitor α-synuclein toxicity and the ability of nac deletion to rescue α-synuclein cytotoxicity (n = 3). (TIF) [file pone.0303008.s002.tif]

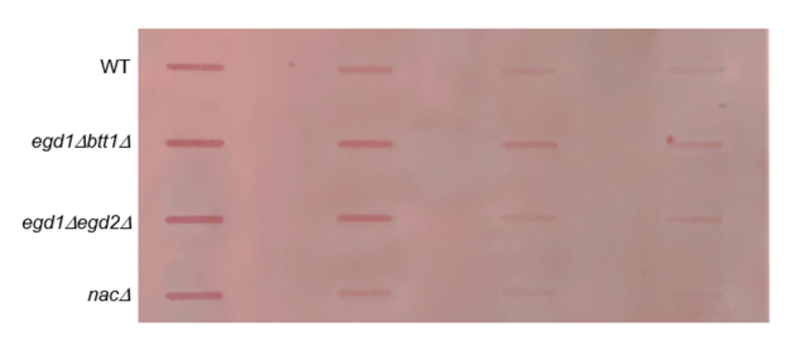

Supplement: S3 Fig — WT and NAC deletion strains expressing Gal1-FLAG-htt25Q-CFP (not shown) or Gal1-FLAG-htt103Q-CFP constructs were grown in selective media in the presence of 2% galactose for 6 hours prior to Filter Trap Assay and Western blotting for FLAG. After developing 2μ filter was soaked in Ponceau staining to show total protein load. All strains are expressing Gal1-FLAG-htt103Q-CFP. (TIF) [file pone.0303008.s003.tif]

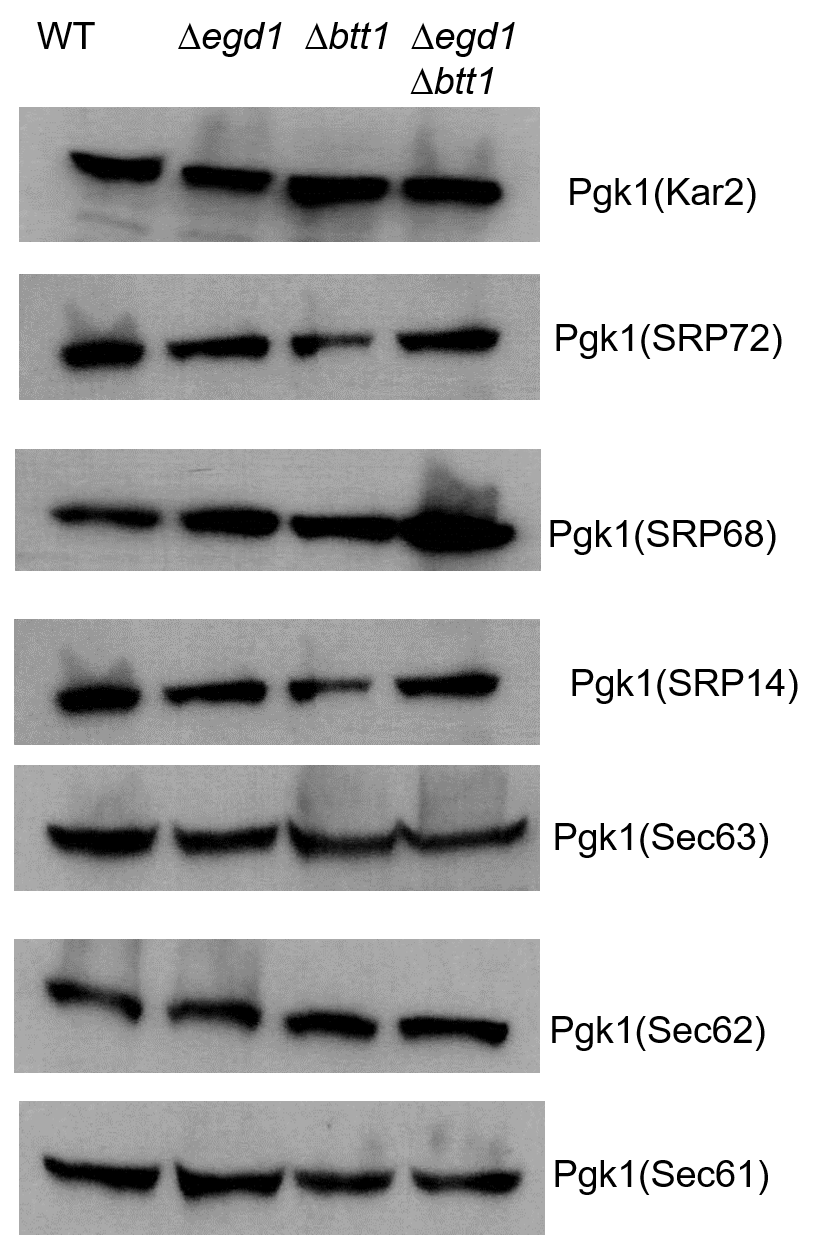

Supplement: S4 Fig — The quantitation of the SRP72/Pgk1 and SRP14/Pgk1 ratios were performed using the same Pgk1 blot as the blot was cut into halves to probe for the original proteins (unlike for the other targeted proteins). Representative images shown here. (TIF) [file pone.0303008.s004.tif]

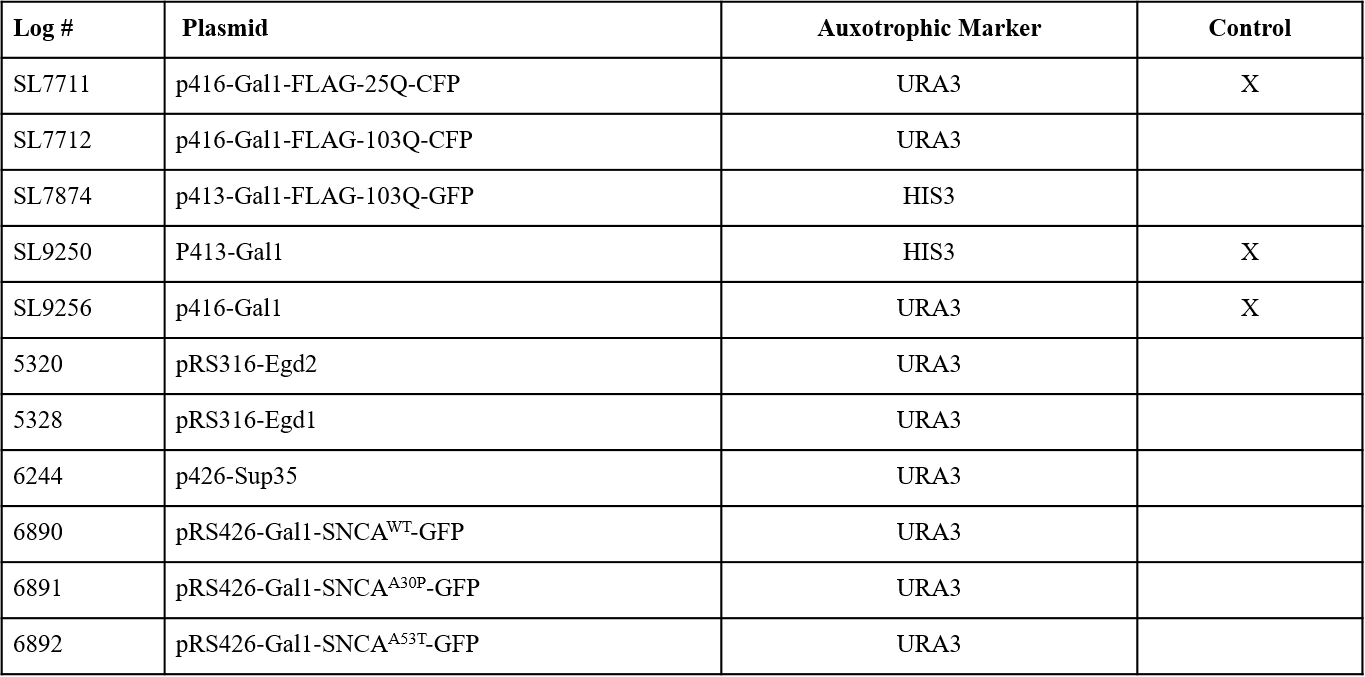

Supplement: S1 Table — The left most column indicates the True Lab log number of each plasmid, the second from the left column contains the plasmid names, the third from the left column indicates the auxotrophic marker of each plasmid, and the right most column identifies the control plasmids. (TIF) [file pone.0303008.s006.tif]

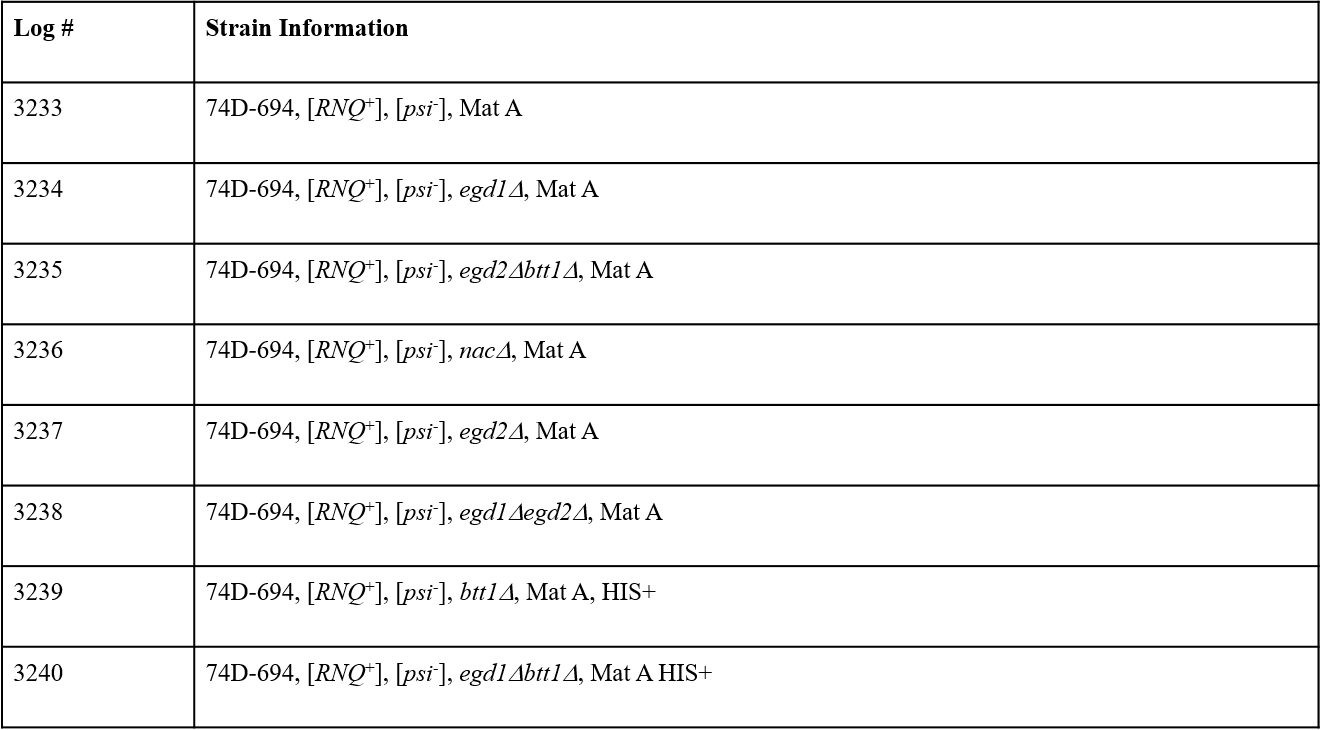

Supplement: S2 Table — The left most column indicates the True Lab log number of each strain and the right column contains relevant strain information. (TIF) [file pone.0303008.s007.tif]
